# Curcuminoid supplementation alters proteomic profiles and clinical outcomes in canine primary epilepsy: A pilot study

**DOI:** 10.1016/j.vas.2026.100906

**Published:** 2026-10-20

**Authors:** Boonrat Chantong, Nutnicha Prakaikowit, Wanita Sangapol, Sirakarn Chatampaiwong, Sekkarin Ploypetch, Sataporn Phochantachinda, Duangthip Chatchaisak

**Affiliations:** a Department of Pre-Clinic and Applied Animal Science, Faculty of Veterinary Science, Mahidol University, Nakhon Pathom, Thailand; b Faculty of Veterinary Science, Mahidol University, Nakhon Pathom, Thailand; c Department of Clinical Sciences and Public Health, Faculty of Veterinary Science, Mahidol University, Nakhon Pathom, Thailand

**Keywords:** Canine epilepsy, Curcuminoids, Mammalian target of rapamycin pathway, One-carbon metabolism, Proteomics

## Abstract

**Background:**

In dogs, primary epilepsy syndrome (PES) requires lifelong pharmacological management. Curcuminoids possess anti-inflammatory and antioxidant properties with potential neuroprotective effects. This pilot study preliminarily evaluated the clinical efficacy and molecular mechanisms of curcuminoid supplementation in canine PES.

**Methods:**

In this prospective, open-label pilot study, eight dogs with PES on stable AED therapy received adjunctive oral curcuminoid supplementation (10-20 mg/kg/day) for 60 days. Clinical response, serum GSH/GSSG ratio (DTNB-glutathione reductase recycling assay), serum cytokine concentrations (ELISA), and global serum proteomic profiles (nano-LC-MS/MS) were assessed.

**Results:**

Clinical response, defined as a reduction in seizure frequency (3/8) or decreased severity (1/8) was observed in 4 of 8 dogs (50%). Curcuminoid supplementation significantly increased the GSH/GSSG ratio (*P* = 0.046) and induced distinct proteomic changes: downregulation of mammalian target of rapamycin (mTOR) pathway components (Ras-related GTP-binding protein C and regulatory-associated protein of mTOR) and Toll-like receptor 2 (TLR2) signaling and upregulation of one-carbon metabolism (methylenetetrahydrofolate dehydrogenase) and serine/glycine/threonine metabolism (phosphoserine aminotransferase 1) enzymes.

**Conclusion:**

Curcuminoid supplementation was safe, well tolerated, and demonstrated a clinical response rate of 50% in dogs with PES. These findings provide preliminary molecular evidence of enhanced antioxidant capacity, mTOR/TLR2 modulation, and metabolic reprogramming. As pilot observations, definitive neuroprotective conclusions require confirmation in larger controlled trials with direct neurological endpoints. Nonetheless, these data provide a molecular rationale for curcuminoids as adjunctive therapy in canine epilepsy.

## 1 Introduction

Epilepsy is one of the most prevalent chronic neurological disorders in veterinary medicine, affecting approximately 0.5%–5.0% of dogs (Ekenstedt & Oberbauer, 2013; Patterson, 2013). It is characterized by recurrent, unprovoked seizures, which significantly impair the quality of life and impose substantial emotional and financial burdens on owners. Standard antiepileptic drugs (AEDs), including phenobarbital and potassium bromide, remain the cornerstone of treatment; however, they fail to achieve adequate seizure control in 20%–30% of patients (Charalambous et al., 2024; Patterson, 2013; Potschka et al., 2023). Moreover, the chronic administration of conventional AEDs is frequently limited by adverse systemic effects (Potschka et al., 2023). As such, there is a need for alternative or adjunctive agents with improved safety profiles and complementary mechanisms of action (Kimram et al., 2025; Razavi et al., 2026).

According to recent pathophysiological studies, epilepsy extends beyond neuronal hyperexcitability to involve complex molecular cascades, including oxidative stress and neuroinflammation (Fabisiak & Patel, 2022; Parsons et al., 2022). Seizure activity induces mitochondrial dysfunction and excessive reactive oxygen species (ROS) production while depleting essential antioxidants, including glutathione (GSH). Concurrently, the upregulation of proinflammatory cytokines, including interleukin-1 beta (IL-1β) and tumor necrosis factor-α (TNF-α), compromises neuronal integrity. These molecular alterations create a self-perpetuating cycle that lowers the seizure threshold and promotes further epileptogenesis (Hamdy et al., 2020; Mehla et al., 2010). Consequently, therapeutic strategies that specifically target oxidative and inflammatory pathways represent promising approaches for neuroprotection and seizure management (Singh et al., 2025).

Curcuminoids, the primary bioactive polyphenols derived from *Curcuma longa* (turmeric), have attracted considerable attention for their multifaceted therapeutic effects, including potent antioxidant, anti-inflammatory, and neuroprotective properties. Among them, curcumin is the most pharmacologically active and extensively studied (Khatoon & Kalam, 2025; Younis & Mohamed, 2025). Considering their favorable safety profile and ability to modulate complex biological pathways, curcuminoids represent potential adjuncts in epilepsy management by targeting the underlying oxidative and inflammatory processes (Dhir, 2018; Razavi et al., 2026). However, despite promising preclinical evidence, few clinical studies have evaluated the efficacy of curcuminoids in naturally occurring canine epilepsy. Moreover, the molecular mechanisms underlying their potential therapeutic effects remain unknown.

In experimental epilepsy models, curcuminoids demonstrate multimodal neuroprotective activity by scavenging ROS and upregulating endogenous antioxidant defenses, including superoxide dismutase and GSH, via nuclear factor erythroid 2-related factor 2 (Nrf2) pathway activation, thereby reducing oxidative stress and seizure-induced neuronal damage (Dhir, 2018; H. Kaur et al., 2014; Kaur et al., 2015; Khatoon & Kalam, 2025). Curcuminoids also improve mitochondrial function and modulate key signaling cascades, including nuclear factor-κB (NF-κB), MAPK, and mammalian target of rapamycin (mTOR) pathways that are critically implicated in epileptogenesis, collectively downregulating proinflammatory mediators (e.g., IL-1β, TNF-α, and cyclooxygenase-2) (Drion et al., 2016; Garodia et al., 2023; H. Kaur et al., 2014; Hamdy et al., 2020; Kaur et al., 2015). Furthermore, curcumin modulates microglial activity by interacting with protein tyrosine phosphatase receptor type Z1, regulating the phosphorylation of proteins linked to inflammation control and neuronal repair (Zhang et al., 2025b). Collectively, curcumin’s potent suppression of neuroinflammation and glial activation, along with the rescue of cognitive deficits, positions it as a promising multi-target adjunct therapy for epilepsy (H. Kaur et al., 2014; Kaur et al., 2015).

According to converging evidence from preclinical animal model studies, curcumin administration reproducibly attenuates seizure frequency and severity, prolongs latency to seizure onset, and confers neuroprotection against hippocampal injury. Using the increasing current electroshock paradigm in mice, Bharal et al. (2008) reported that curcumin, administered orally at 100 mg/kg (both acutely and following 21-day chronic dosing), produced a statistically significant elevation in the seizure threshold, providing direct pharmacological evidence for intrinsic anticonvulsant activity (Bharal et al., 2008). In a pentylenetetrazol-induced rat model of epilepsy, Hamdy et al. (2020) further elucidated the underlying neuroprotective mechanisms of curcumin. They demonstrated that curcumin attenuated seizure-associated neuronal apoptosis and modulated the Wnt/β-catenin and autophagy signaling cascades, thereby implicating these pathways as key molecular targets of curcumin’s antiseizure action (Hamdy et al., 2020). Advances in formulation, including micronization and nanotechnology, have enhanced the bioavailability and brain penetration ability of curcumin, resulting in comparable anticonvulsant effects to those of conventional AEDs in rodent and zebrafish models (Bertoncello et al., 2018; Mehla et al., 2010).

Mass-spectrometry-based proteomics enables the comprehensive mapping of molecular alterations associated with epilepsy and therapeutic interventions (Banote et al., 2021; Liu et al., 2010; Ma et al., 2024), which can simultaneously identify and quantify protein networks regulating oxidative stress, neuroinflammation, and synaptic signaling, thereby revealing treatment response biomarkers and mechanisms underlying therapeutic efficacy (Bitsika et al., 2016; Tse et al., 2019; Walker et al., 2016). Previous studies have used plasma proteomics to identify epilepsy-associated protein signatures across species, including causal candidates through Mendelian randomization (Fu et al., 2025), seizure-linked immune and coagulation clusters (Ashtiani et al., 2025), and upregulated complement components and apolipoproteins in temporal lobe epilepsy (Glazyrin et al., 2024). In canine refractory epilepsy, 40 out of 155 plasma proteins were differentially expressed—with epilepsy-exclusive ceruloplasmin and complement factor H, absence of gelsolin, elevated haptoglobin, and markedly increased amyloid-beta 42 (Aβ42) implicating neuroinflammatory and coagulation pathways as central disease drivers (Phochantachinda et al., 2023). However, these dysregulated proteins align directly with curcuminoids’ anti-inflammatory, antioxidant, neuroprotective, and amyloid-modulating mechanisms, providing a strong biological rationale for proteomic-guided monitoring of curcuminoid supplementation in epileptic dogs (Phochantachinda et al., 2023; D. Zhang et al., 2025a). Therefore, serum proteomics could enable simultaneous tracking of curcuminoid-induced normalization across haptoglobin, ceruloplasmin, complement factor H, alpha-2-macroglobulin, gelsolin, and Aβ42, offering mechanistic insights beyond seizure-frequency assessment alone (Ashtiani et al., 2025; Glazyrin et al., 2024). Collectively, the conserved proteomic signatures of epilepsy across humans and dogs support plasma/serum proteomics as a feasible, minimally invasive, and mechanistically informative tool for investigating the effects of curcuminoids on canine epilepsy.

However, despite extensive preclinical evidence supporting the anticonvulsant and neuroprotective effects of curcumin, the specific molecular alterations and proteomic landscapes associated with its therapeutic action in canine epilepsy remain largely unexplored. Therefore, this study evaluated the clinical efficacy and molecular mechanisms of curcuminoid supplementation in dogs with primary epilepsy syndrome (PES) through integrated clinical assessment and plasma proteomic profiling. Curcuminoid supplementation was hypothesized to attenuate the frequency and severity of seizures while modulating oxidative stress biomarkers and serum protein networks associated with neuroinflammation and neuroprotection.

## 2 Materials and methods

### 2.1 Study design and ethical approval

This prospective, open-label pilot study was conducted at Prasu Arthorn Animal Hospital, Faculty of Veterinary Science, Mahidol University, Nakhon Pathom, Thailand. The study protocol was approved by the Mahidol University Institute Animal Care and Use Committee of the Faculty of Veterinary Science (Protocol No. MUVS-2020-08-37). Written informed consent was obtained from all dog owners prior to enrollment.

### 2.2 Animals

Eight client-owned dogs with PES were enrolled alongside five healthy adult dogs (aged 1–6 years; mixed breeds and purebreds; no history of neurological disease, seizures, or systemic illness) recruited from the same institution as a proteomic reference control group. Control dogs underwent a single blood collection using the same protocol and did not receive curcuminoid supplementation. This group was used solely for proteomic comparison (Section 2.8) to identify proteins differentially expressed in the epileptic state (adults vs. C0) and to distinguish disease-state changes from treatment-induced alterations (C60 vs. C0).

The required sample size was calculated using GPower 3.1 software (Heinrich Heine University Düsseldorf, Germany) with the following parameters: paired t-test (two-tailed), α = 0.05, power (1-β) = 0.80, and an anticipated effect size (Cohen’s d) of 1.0 based on previous studies demonstrating the large effects of antioxidant interventions on the GSH/oxidized glutathione (GSSG) ratio (Alizadeh & Kheirouri, 2019; Faul et al., 2007; Roohi et al., 2017). This calculation yielded a minimum required sample size of eight dogs, with an actual achieved power of 80.82%.

It should be noted that the sample size was calculated for the primary biochemical endpoint (GSH/GSSG ratio) and does not power the proteomic analysis, which is exploratory and hypothesis-generating. Multi-layered statistical filtering (ANOVA, FDR correction, and VIP scoring) was applied to minimize false discoveries in the high-dimensional dataset. Validation in larger, prospectively powered cohorts is required.

The inclusion criteria were as follows: (1) diagnosis of idiopathic/primary epilepsy based on the International Veterinary Epilepsy Task Force criteria (Berendt et al., 2015), (2) history of recurrent seizures (≥2 seizures within the previous six months), (3) age between 1 and 10 years, (4) stable AED regimen for at least 30 days prior to enrollment, and (5) owner compliance with seizure diary maintenance. Meanwhile, the exclusion criteria were as follows: (1) structural brain lesions identified by magnetic resonance imaging, (2) metabolic or toxic causes of seizures, (3) concurrent systemic diseases, and (4) administration of immunosuppressive or anti-inflammatory medications within 30 days of enrollment.

The AED regimens of the enrolled dogs (drug name, dose, and duration of stable therapy prior to enrollment) are summarized in Table 1. All dogs were receiving phenobarbital (2.0–5.0 mg/kg, twice daily) as the primary AED. No changes to AED therapy were made during the study period. All owners were instructed to maintain their dogs' existing commercial diets unchanged throughout the study period.

**Table 1:** Signaling and clinical characteristics of dogs with primary epilepsy syndrome enrolled in the curcuminoid supplementation study.

Dog No.	Breed	Sex	Age (years)	Weight change (kg)	Seizure type	Phenobarb (PB) dose (mg/kg q12h)	Seizure observed during PB + curcuminoids (60 days)				Clinical outcome (60 days)
							Baseline seizure Frequency	Baseline seizure Duration and pattern	Day 60 seizure Frequency	Day 60 seizure Duration and pattern	
1	Mixed breed	F	4	31.6 -31.2	PES	2.9	1 episode/ 1 month	2 mins (generalized)	1 episode/ 2 months	2 mins (generalized)	Decreased frequency
2	Alaskan Malamute	F	3	29.4-28.5	PES	2.1	1 episode/ 1 month	2 mins (focal: left hindlimb)	1 episode/ 2 months	2 mins (focal: left hindlimb)	Decreased frequency
3	Beagle	F	7	13-13.5	PES	4.4	1 episode/ 2 months	3 mins (focal: bilateral facial)	1episode/ 2 months	3 mins (focal: bilateral facial)	Stable
4	Mixed breed	F	9	23-22.8	PES	2.63	1 episode/ 2 months	2 mins (focal: right facial)	1 episode/ 2 months	2 mins (focal: right facial)	Stable
5	Siberian Husky	M	4	31-31	PES	1.93	1 episode/ 2 months	1 min (generalized)	1 episode/ 2 months	1 min (generalized)	Stable
6	Labrador Retriever	M	6	45.6-43.9	PES	1.97	1 episode/ 2 months	2 mins (generalized)	0 episode/ 2 months	None	Decreased frequency
7	Mixed breed	M	10	21-20.5	PES	2.85	1 episode/ 2 months	3mins (generalized: clonic)	1 episode/ 2 months	1 min (generalized: clonic)	Improved symptoms
8	Pomeranian	M	5	9.8-9.8	PES	3.1	1 episode/ 1 month	3 mins (bilateral facial)	1 episode/ 1 month	3 mins (bilateral facial)	Stable

All enrolled dogs underwent baseline clinical examination, complete blood count (CBC), serum biochemistry profile, and neurological examination. The seizure frequency and severity were documented using standardized owner seizure diaries for 30 days prior to intervention (baseline period).

### 2.3 Curcuminoid supplementation protocol

Dogs received oral curcuminoid supplementation at 10–20 mg/kg/day (100-mg capsules; Kaewmangkorn Pharmacy Co., Ltd., Thailand) for 60 consecutive days. The dose was derived from preclinical studies using the body surface area normalization method (Reagan‐Shaw et al., 2008) and is supported by demonstrated safety and tolerability in dogs (Boonyarattanasoonthorn et al., 2022) and anticonvulsant and neuroprotective efficacy in rodent seizure models (Harpreet Kaur et al., 2014; Kiasalari et al., 2013; Saha et al., 2016; Sharma et al., 2010). The supplement was administered with food once daily. Concurrent AED therapy remained unchanged throughout the study period.

### 2.4 Clinical assessment

Clinical response was assessed by comparing seizure characteristics at baseline (day 0 of curcuminoid supplemented seizure dogs, C0) and day 60 (day 60 of post-supplementation, C60). The seizure frequency, severity, and post ictal phase duration were recorded by the owners. Data were collected from owner-maintained records (owner logbooks).

Clinical improvement was defined as a ≥25% reduction in seizure frequency, a ≥25% increase in the inter-ictal period, or a transition from cluster seizures to single events. Additional indicators included improved seizure symptomatology, characterized by reduced clonic movements or shorter duration, and lessened post ictal severity or recovery time.

### 2.5 Sample collection

Blood samples (5 mL) were collected via cephalic or jugular venipuncture at C0 (Day 0) and C60 (Day 60) following a 12-h fast. The ethylenediaminetetraacetic acid (EDTA) blood samples were stored at 4°C, and the CBCs were measured using an animal blood counter (Horiba Medical, Montpellier, France) within 4 h. Plasma was separated by centrifugation (3,000 × g, 10 min, 4°C) within 30 min of collection. Serum samples were allowed to clot for 30 min at room temperature before centrifugation (3,000 × g, 15 min, 4°C). All samples were stored at −80°C until analysis. Plasma was used for routine biochemistry (Section 2.6.2). Serum was used for GSH/GSSG ratio (Section 2.6.3), malondialdehyde concentration (Section 2.6.4), cytokine (IL-6, IL-10) ELISA (Section 2.6.5), and global proteomic profiling by nano-LC–MS/MS (Section 2.7).

### 2.6 Biochemical and hematological analyses

#### 2.6.1 Routine blood chemistry

Hepatic function markers (alkaline phosphatase [ALP], alanine aminotransferase [ALT], aspartate aminotransferase [AST], and total bilirubin), renal function markers (blood urea nitrogen [BUN] and creatinine), metabolic markers (glucose, calcium, total protein, albumin, and globulin), electrolytes (sodium, potassium, and chloride), and acid–base balance (total carbon dioxide) were measured using an automated blood chemistry analyzer (VETSCAN VS2; Zoetis Inc., Parsippany, NJ, USA).

#### 2.6.2 Complete blood count

CBC analysis was performed at baseline (Day 0) and at the end of the intervention period (Day 60). The following hematological parameters were evaluated: white blood cell count (WBC), differential leucocyte counts (monocytes, neutrophils, lymphocytes, eosinophils, basophils, and band neutrophils), red blood cell count (RBC), haemoglobin concentration (Hb), haematocrit (Hct), mean corpuscular volume (MCV), mean corpuscular haemoglobin (MCH), mean corpuscular haemoglobin concentration (MCHC), platelet count, red cell distribution width (RDW), and plasma protein. Results were interpreted against established canine reference intervals, and samples were qualitatively assessed as adequate, increased, or decreased relative to these ranges.

#### 2.6.3 GSH/GSSG ratio

Serum reduced glutathione (GSH) and oxidized glutathione (GSSG) concentrations were measured using the 5,5′-dithiobis-(2-nitrobenzoic acid) (DTNB)/glutathione reductase enzymatic recycling method (Rahman et al., 2006; Tipple & Rogers, 2012). Total glutathione (GSH + GSSG) was quantified by monitoring DTNB reduction at 412 nm in the presence of glutathione reductase and NADPH; GSSG was determined after masking GSH with 2-vinylpyridine. The GSH/GSSG ratio was calculated as an index of cellular redox status. All samples were analyzed in duplicate, and mean values were used for statistical analysis.

#### 2.6.4 Malondialdehyde concentration

Serum malondialdehyde (MDA) concentration was measured as an index of lipid peroxidation using the thiobarbituric acid reactive substances (TBARS) assay (Gheita & Kenawy, 2014). Serum samples were mixed with trichloroacetic acid and thiobarbituric acid reagent, heated at 95°C for 15 min, cooled, and centrifuged. Absorbance of the resulting pink chromogen was measured spectrophotometrically at 532 nm, and MDA concentration was calculated from a 1,1,3,3-tetramethoxypropane standard curve and expressed as nM.

#### 2.6.5 Cytokine concentrations

Serum concentrations of interleukin-6 (IL-6) and interleukin-10 (IL-10) were determined independently by canine-specific sandwich enzyme-linked immunosorbent assay (ELISA) using the Canine IL-6 ELISA Kit (catalog no. ab193686; Abcam, Cambridge, UK) and the Canine IL-10 ELISA Kit (catalog no. ab193685; Abcam, Cambridge, UK), respectively. Each assay employs matched capture and detection antibodies raised against the respective canine cytokine, with a horseradish peroxidase (HRP)-conjugated secondary antibody and 3,3′,5,5′-tetramethylbenzidine (TMB) as the chromogenic substrate; optical density was measured at 450 nm. All samples were assayed in duplicate according to the manufacturer's instructions, and cytokine concentrations were interpolated from kit-specific standard curves. Results were expressed as ng/mL.

### 2.7 Proteomic analysis

#### 2.7.1 In-solution digestion

Serum proteins were purified using the Pierce Albumin/IgG Removal Kit (catalog no. 89875; Thermo Fisher Scientific, Waltham, MA, USA) according to the manufacturer's instructions. Subsequently, the obtained protein pellets were dissolved in 8 M urea, and the protein concentration was measured using Bradford assay (Bio-Rad Protein Assay, Bio-Rad Laboratory, CA, USA). For each sample (containing 30 μg of protein), reduction was performed with 100 mM dithiothreitol in 100 mM tetraethylammonium bromide (TEAB; 37°C, 30 min), followed by an alkylating buffer of 100 mM iodoacetamide in 100 mM TEAB (30 min at 25°C, 30 min). The reaction was quenched (25°C, 15 min) before the sample was digested using trypsin gold (mass spectrometry [MS] grade; Promega, USA) (37°C, 16 h). Then, the purified peptides were dried using a nitrogen evaporator, stored at −80°C, and reconstituted in 0.1% formic acid before quantification with a NanoDrop 1000 instrument (Thermo Fisher Scientific, Bremen, Germany).

#### 2.7.2 Nano-liquid chromatography–tandem MS

The extracted serum peptides (1 µg) were analyzed using a nano-liquid chromatography–tandem MS system (Dionex UltiMate 3000 RSLCnano System, Thermo Scientific) and a quadrupole time-of-flight compact mass spectrometer (Bruker, Germany). Separation was performed on a C18 column with a constant flow rate of 300 nL/min at 60°C using a linear gradient of mobile phase A (0.1% formic acid) and mobile phase B (0.08% formic acid in 80% acetonitrile). MS was performed in the positive ion mode with a specific mass range of 150–2,200 m/z.

#### 2.7.3 Protein identification and quantification

Raw MS data were processed using MaxQuant software (v1.6.2.10) (Cox & Mann, 2008; Tyanova et al., 2016) equipped with the Andromeda search engine (Cox et al., 2011). Data were searched against the *Canis lupus familiaris* proteomic database (www.uniprot.org), and the analysis was set to 1 for the label-free approach. The oxidation of methionine and acetylation of the N-terminus were set as variable modifications, whereas the carbamidomethyl modification of cysteine was set as a fixed modification. The false discovery rate was set to 1% at the peptide level, with a TOF MS/MS match tolerance of 0.5 Da.

The resulting label-free quantification was performed and imported into Perseus software (v1.6.8.0) (https://www.maxquant.org) for statistical analysis (Tyanova et al., 2016). Differentially expressed proteins were identified using a two-sample test, with statistical significance being considered at *P* < 0.05. The proteomics data are publicly available through the ProteomeXchange Consortium via the PRIDE (Deutsch et al., 2026; Jarnuczak & Vizcaíno, 2017) partner repository (dataset PXD076578).

### 2.8 Variable importance in projection analysis

Differentially expressed proteins were analyzed using MetaboAnalyst 6.0 (http://www.metaboanalyst.ca) (Ewald et al., 2024). Data filtering, missing value handling, and normalization were performed in MetaboAnalyst 6.0. To satisfy parametric statistical assumptions (normality and variance homogeneity), data were subjected to auto-scaling (and/or log transformation), which effectively reduced skewness and stabilized feature variance across sample groups. For multigroup comparisons (C0, C60, and adults), statistical significance was defined by one-way analysis of variance (ANOVA) followed by Tukey’s post hoc test. Statistical significance was defined at *p* < 0.01 (post-hoc *p* < 0.05) with False Discovery Rate (FDR) control (*q*-value < 0.05). Effect sizes were represented by Fold Change (FC) and log_2_FC values to indicate the magnitude of difference for significant features. To identify key contributors to the discrimination between specific groups (C60 vs. C0 and adults vs. C0), a variable importance in projection (VIP) threshold of >1.0 was applied. Multivariate analysis was performed using partial least squares discriminant analysis (PLS-DA) to assess group separation. Overlap among the significant protein sets, specifically those derived from ANOVA, and the high-VIP selections from both pairwise comparisons were visualized via Venn diagrams (Bardou et al., 2014). Candidate proteins were prioritized based on their presence in the intersection of all three statistical filters.

### 2.9 Pathway enrichment analysis

Protein–protein and protein–chemical interaction networks were mapped using the STITCH database (v5.0; EMBL; http://stitch.embl.de; accessed December 7, 2025) (Szklarczyk et al., 2016). This analysis integrated the identified differentially expressed proteins to explore distinct patterns of upregulation and downregulation within the context of curcumin–target interactions. Pathway analysis was performed using the Kyoto Encyclopedia of Genes and Genomes database to investigate protein interactions with curcumin. Protein functions were identified through the UniProt database (www.uniprot.org) (Zaru et al., 2023).

### 2.10 Statistical analysis

Statistical analyses were performed using GraphPad Prism and R software. Clinical data (seizure frequency and severity) were compared between baseline and intervention periods using paired t-tests or Wilcoxon signed-rank tests as appropriate. GSH/GSSG ratios were compared between time points using paired t-tests. The results are presented as mean ± standard deviation or median (interquartile range) as appropriate. Statistical significance was defined as *P* < 0.05.

## 3 Results

### 3.1 Study population characteristics

Eight client-owned dogs (four males and four females) with confirmed PES were enrolled. The cohort had a median age of 5.75 years (range 3–10 years) and included diverse breeds: Mixed Breed (n = 3), Alaskan Malamute (n = 1), Beagle (n = 1), Siberian Husky (n = 1), Labrador Retriever (n = 1), and Pomeranian (n = 1) (Table 1). In addition to maintenance phenobarbital treatment (1.93 – 4.4 mg/kg q12h), all subjects were supplemented with 100 mg/day of curcuminoids for a duration of 60 days.

### 3.2 Clinical response to curcuminoid supplementation

Clinical response (≥25% reduction in seizure frequency and/or ≥1 grade reduction in seizure severity) was observed in four dogs (50%) (Table 1). Within responder group, three dogs (No. 1, 2, and 6) exhibited decreased seizure frequency, and one dog (No. 7) showed improved seizure symptoms with reduced clonic movements. Notable clinical improvements included a transition from cluster seizures to single, non-cluster events and prolonged inter-ictal periods. In the four non-responder dogs (No. 3,4, 5, and 8), seizure frequency and severity remained stable compared with baseline, with no significant changes being observed. No dogs experienced worsening of seizure control during the intervention period.

Eight client-owned dogs (four males and four females; age: 3–10 years; body weight: 9.6–45.6 kg) received 100 mg of curcuminoid supplementation daily for 60 days. Clinical response (decreased seizure frequency or improved symptom severity) was observed in four dogs (50%).

### 3.3 Safety and tolerability

Curcuminoid supplementation was well tolerated in all dogs. No serious adverse events were reported during the 60-day intervention period. Moreover, no clinically significant changes in serum biochemistry parameters (Table 2) or CBC (Table 3) were observed on day 60 compared with baseline. No clinically significant hepatotoxicity, nephrotoxicity, or other adverse effects were observed during the study period. Although serum ALP was elevated, this is consistent with phenobarbital-induced hepatic microsomal enzyme induction rather than hepatotoxicity, supported by the absence of concurrent elevations in bilirubin, ALT, or AST and no clinical signs of liver disease (Aitken et al., 2003; Gaskill et al., 2005; Gaskill et al., 2004; Müller et al., 2000).

**Table 2:** Comparison of serum biochemistry parameters between baseline (day 0) and post-supplementation (day 60) in dogs with primary epilepsy syndrome receiving curcuminoid supplementation.

Parameters		Curcuminoid supplementation						
		Day 0			Day 60			*P*-value
	Reference interval	Median	Range	SD	Median	range	SD	
Alkaline phosphatase (U/L)	23-212	994.00	32–1374	565.33	922	40–1266	518.87	0.674
Alanine aminotransferase (U/L)	10-125	50.50	41–146	40.51	50	39–143	34.40	0.779
Aspartate aminotransferase (U/L)	10–50	34.50	22–41	6.02	33	25–37	3.60	0.107
Total bilirubin (mg/dL)	0–0.9	0.30	0.3–0.4	0.35	0.3	0.3–0.6	0.12	0.197
Blood urea nitrogen (mg/dL)	7–27	14.00	9 –17	2.60	13	9–16	2.07	0.228
Creatinine (mg/dL)	0.5–1.8	1.05	0.6–1.30	0.24	1.05	0.8–1.2	0.15	0.914
Glucose (mg/dL)	74–143	96.00	28–79	9.48	95.5	33–7	10.27	0.672
Calcium (mg/dL)	9–11.3	10.25	9.6–10.9	0.44	10.4	9.7–10.9	0.41	0.611
Total protein (g/dL)	5.2–8.2	6.50	6.1–7.6	0.53	6.6	6.1–7.9	0.57	0.944
Albumin (g/dL)	2.3 – 4.5	3.75	3.10–3.90	0.33	3.55	3–4.1	0.33	0.944
Globulin (g/dL)	2.5 – 4.5	2.95	2.40–4.50	0.67	2.95	2.5–3.8	0.54	0.527
Sodium ion (mmol/L)	144 – 160	147.00	141–149	2.60	147.00	143–152	3.23	0.089
Potassium ion (mmol/L)	4.1 – 5.8	4.85	4.50–5.30	0.24	4.60*	4.3–5	0.23	0.047
Chloride ion (mmol/L)	105 – 122	113.00	110.00–115.00	2.00	111.00	106.00–116.00	3.91	0.091
Total carbon dioxide (mmol/L)	18 - 27	23.50	18.00–26.00	2.71	22.00	20.00–27.00	2.53	0.798

**Table 3:** Complete blood count parameters of dogs before (Day 0) and after (Day 60) curcuminoid supplementation. Values are reported for individual animals across eight breeds. Reference ranges represent established canine hematological intervals.

Parameters	Unit	Reference Range	Dog No. 1		Dog No. 2		Dog No. 2		Dog No. 3		Dog No. 4		Dog No. 5		Dog No. 6		Dog No. 7	
			Curcuminoid supplementation		Curcuminoid supplementation		Curcuminoid supplementation		Curcuminoid supplementation		Curcuminoid supplementation		Curcuminoid supplementation		Curcuminoid supplementation		Curcuminoid supplementation	
			Day 0	Day 60	Day 0	Day 60	Day 0	Day 60	Day 0	Day 60	Day 0	Day 60	Day 0	Day 60	Day 0	Day 60	Day 0	Day 60
WBC	10^3/uL	6-17	13.64	16.27	11.4	14.24	9.13	9.12	8.33	8.51	14.65	12.84	8.18	9.34	13.22	12.76	15.92	13.19
Monocyte	%	3-10	5	4	4	6	2 (L)	3	2 L	2 L	3	8	3	7	4	7	4	6
Neutrophil	%	60-77	72	67	67	77	87 (H)	70	79 H	71	77	70	78 H	68	73	71	69	79 H
Lymphocyte	%	12-30	21	20	22	13	11 (L)	23	19	25	16	22	18	25	22	17	27	14
Eosinophil	%	2-10	2	9	7	4	0 (L)	4	0 L	2	4	0 (L)	1 L	0 L	1 L	5	0 L	1 L
Basaphil	%	Rare	0	0	0	0	0	0	0 H	0 H	0	0	0	0	0	0	0	0
Band neutrophil	%	0-3	0	0	0	0	0	0	0	0	0	0	0	0	0	0	0	0
RBC	10^6/uL	5-9	5.64	5.37	6.31	5.59	5.54	5.56	7.42	7.45	6.7	6.03	5.51	5.92	5.7	5.23	5.38	6.29
Hb	g/dL	10-18	13.8	13.6	14.9	13.6	13.5	13.9	16.7	17.2	16.9	15.3	14.9	14.8	14.5	12.8	14.3	16.3
Hct	%	35-55	40.6	38.2	44.8	39.5	39.5	40.8	49	49.3	50.2	44.4	40.5	42.3	43.8	39.1	41.6	48
MCV	fL	60-77	72.1	71.1	71	70.6	71.3	73.4	66	66.2	75	73.7	73.5	71.4	76.8	74.8	77.4 H	76.4
MCH	pg	20-25	24.5	25.3 H	23.6	24.3	24.4	25	22.5	23.1	25.2 (H)	25.4 (H)	27 H	25	25.4 H	24.5	26.6 H	25.9 H
MCHC	g/dL	32-36	34	35.6	33.3	34.5	34.2	34	34.1	34.9	33.7	34.4	36.8 H	35	33.1	32.7	34.3	33.9
Platelet	10^3/uL	200-500	250	356	204	194 L	554 (H)	555 H	233	215	177 (L)	179 (L)	171	72 L	202	238	274	329
RDW	%	12-15	12.4	11.6 L	11.8 (L)	11.5 L	12.3	11.5 L	12.3	12.2	11.5 (L)	11.5 (L)	13	12.6	12.5	12.1	11.5 L	11.6 L
Plasma protein	g/dL	6-7.5	7.6 (H)	7	8.0 (H)	6	7	6.6	7	6.6	7	6	7	7	9 (H)	7.4	8 H	8.4 H

Data are presented as median (range) and standard deviation (SD). Statistical analysis was performed using the paired Wilcoxon signed-rank test or paired t-test, as appropriate. The serum potassium concentration showed a significant change between timepoints (*P = 0.047). All other parameters remained within the reference ranges with no significant differences.

### 3.4 Oxidative stress and inflammatory markers

Oxidative stress assessment revealed a statistically significant increase in the GSH/GSSG ratio from C0 to C60 (*P* = 0.046), indicating enhanced antioxidant capacity following curcuminoid supplementation (Fig. 1A).

**Fig. 1:**
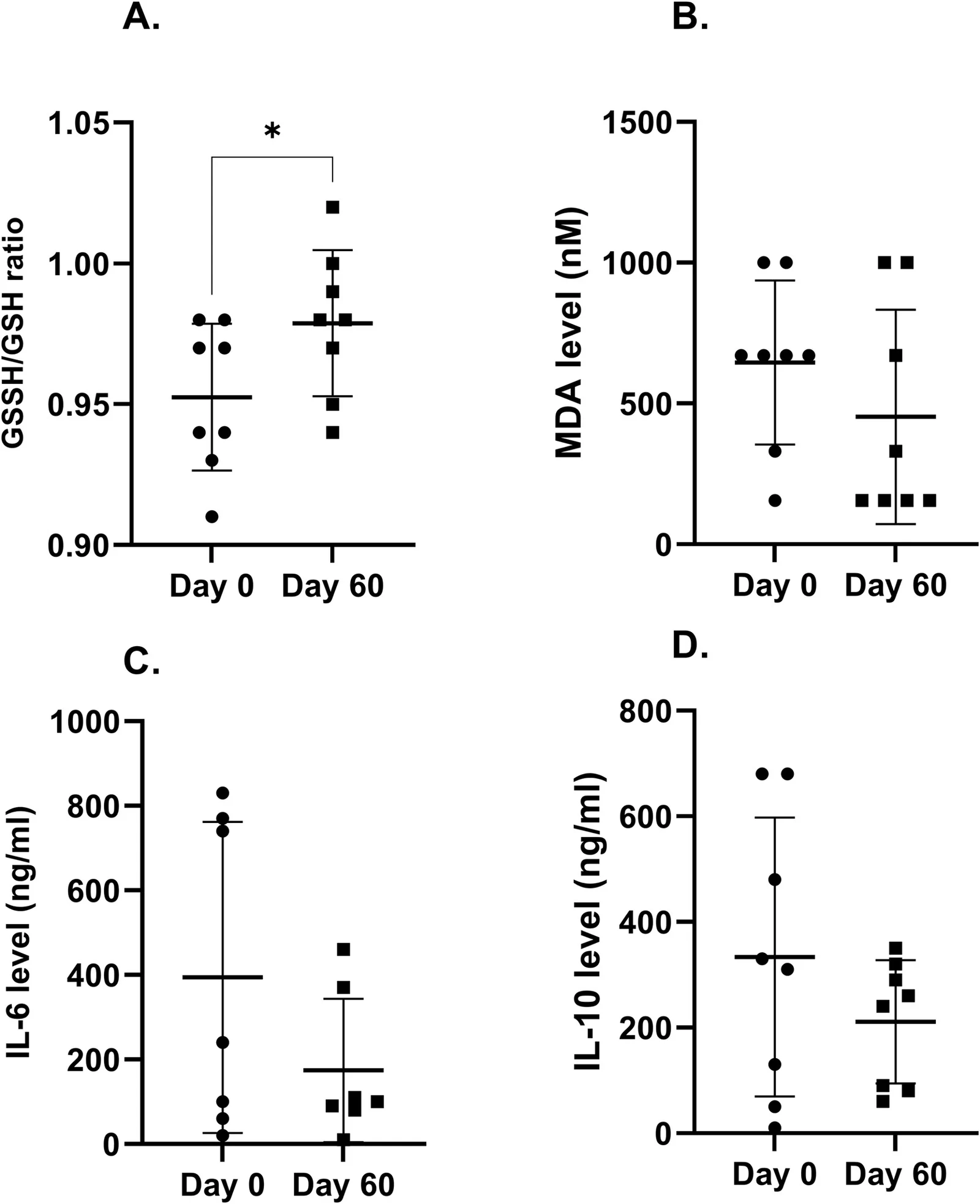
Effects of 60-day curcuminoid supplementation on systemic oxidative stress and inflammatory markers in dogs with primary epilepsy. The box-and-whisker plots illustrate the serum concentrations of key biochemical markers at baseline (day 0, C0) and after 60 days of curcuminoid treatment (day 60, C60). (A) The reduced glutathione to oxidized glutathione (GSH/GSSG) ratio showed a statistically significant increase (*P* = 0.046), indicating an enhancement of systemic antioxidant capacity. No statistically significant differences in (B) malondialdehyde (MDA, a lipid peroxidation marker), (C) interleukin (IL)-6 (a proinflammatory cytokine), or (D) IL-10 (an anti-inflammatory cytokine) were observed between C0 and C60. Data are presented as median and range, with individual data points overlaid. Statistical comparisons were performed using the Wilcoxon signed-rank test. Statistical significance was considered at *P* < 0.05.

MDA, a lipid peroxidation marker, showed no significant change between C0 and C60 (Fig. 1B). Similarly, the serum concentrations of IL-6 (a proinflammatory cytokine) and IL-10 (an anti-inflammatory cytokine) remained stable throughout the intervention period (Fig. 1C and 1D).

### 3.5 Proteomic profiling

#### 3.5.1 Differential proteomic profiling of curcuminoid supplementation

MS-based proteomic analysis identified 4,742 proteins that were significantly altered following curcuminoid supplementation (ANOVA *P*< 0.01, post hoc *P* < 0.05) (Fig. 2). Volcano plot analysis demonstrated that proteins reaching statistical significance were concentrated in regions of high −log_10_(*P*-value), indicating robust proteomic changes.

**Fig. 2:**
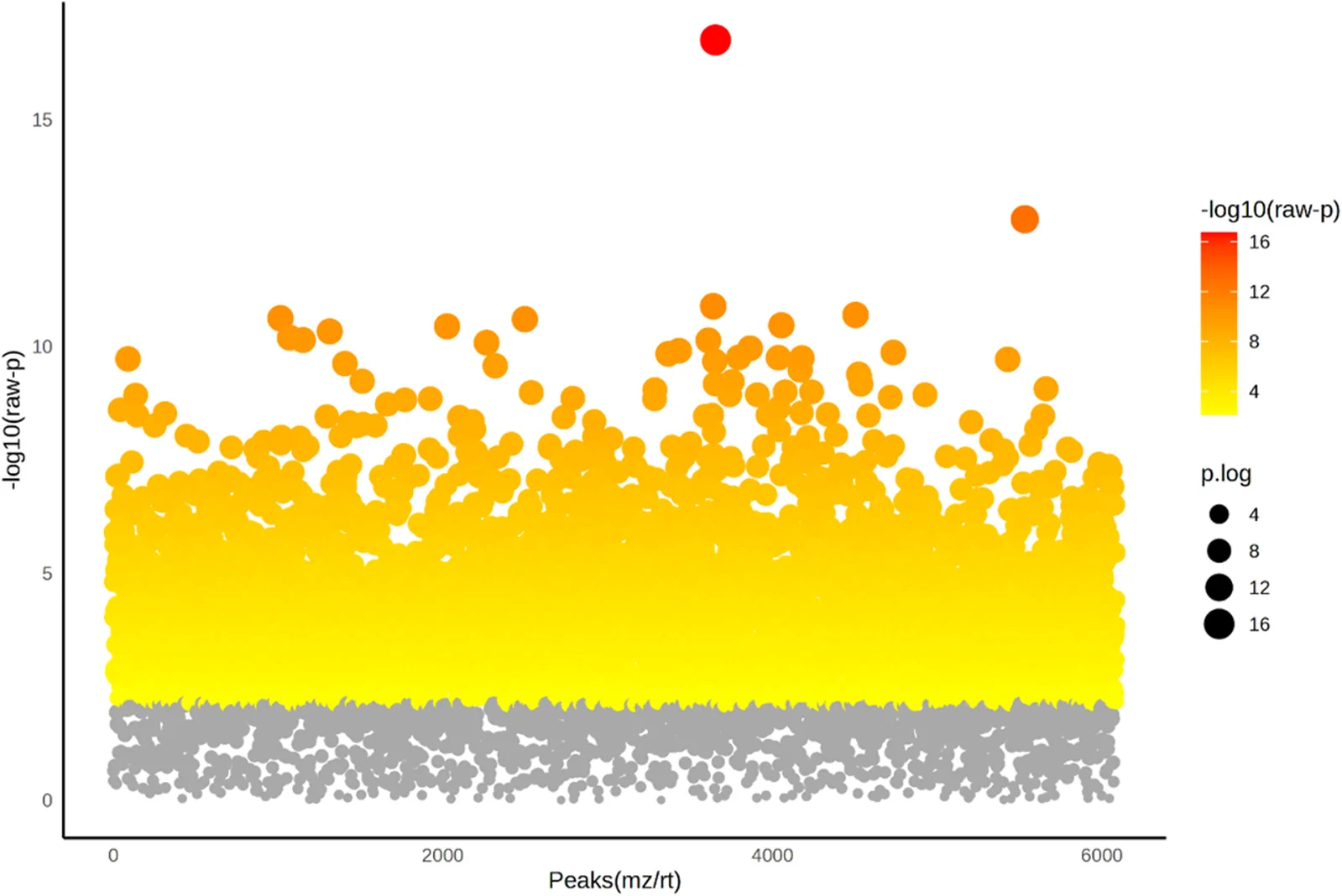
Global proteomic profile of serum peaks in dogs with primary epilepsy. There were a total of 4742 significant proteins, with ANOVA *P* < 0.01 and post hoc *P* < 0.05. A representative volcano plot illustrating the magnitude of protein abundance differences (x-axis: peaks (mz/rt)) and statistical significance (y-axis: −log_10_(raw-p)) derived from proteomic analysis. The color bar (red-to-yellow) and point size indicate statistical significance, highlighting proteins that are highly significant (high-log10(raw-p)). Significant proteins (ANOVA *P* < 0.01) are concentrated in the yellow/orange regions.

Venn diagram analysis of protein overlap across three comparison criteria (C60 vs. C0 VIP>1.0, adults vs. C0 VIP > 1.0, and ANOVA *P* < 0.01 with post hoc *P* < 0.05) revealed seven proteins that were consistently regulated across all groups, including structural proteins albumin and apolipoprotein A1 (Fig. 3). Notably, 20 proteins were uniquely significant in the curcuminoid intervention comparison, suggesting a specific therapeutic proteomic signature.

**Fig. 3:**
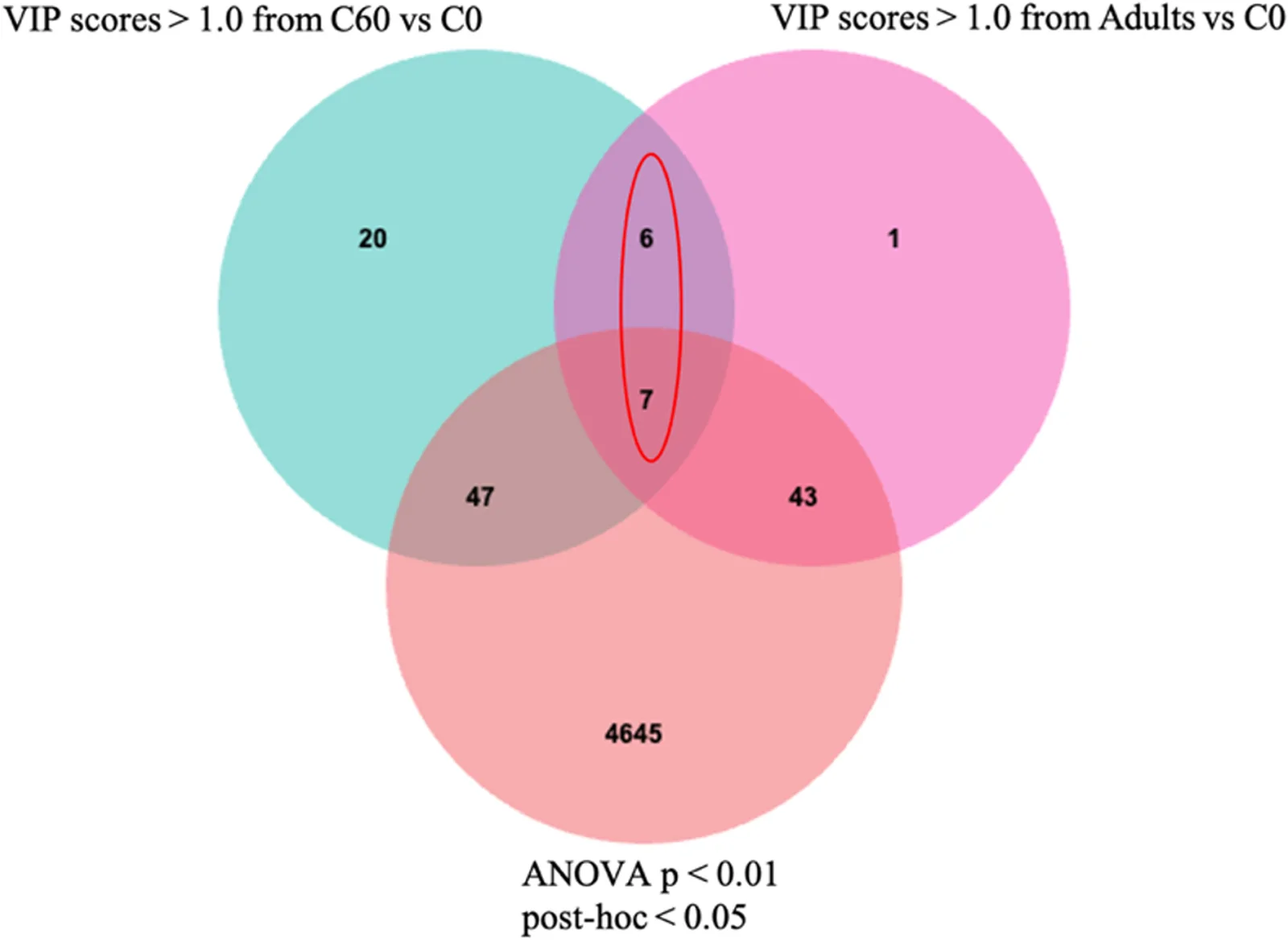
Venn diagram analysis of significant proteins. Venn diagram illustrating the overlap of significantly altered proteins across three comparisons: (1) variable importance in projection (VIP) scores > 1.0 from C60 vs. C0 (curcumin day 60 vs. day 0), (2) VIP scores > 1.0 from adults vs. C0 (adult controls vs. day 0), and (3) proteins significant by ANOVA (*P* < 0.01, post hoc *P* < 0.05). The diagram highlights seven common significant proteins found in all three groups and six significant proteins shared specifically between the C60 vs. C0 and adults vs. C0 groups, emphasizing conserved metabolic and structural proteins affected by the intervention.

#### 3.5.2 PLS-DA and VIP analysis reveal distinct curcuminoid-induced proteomic signatures

VIP analysis identified 80 proteins with scores >1.0 that distinguished post-treatment (C60) samples from baseline (C0) samples, reflecting the most substantial treatment-induced proteomic changes (Fig. 4A). In addition, 57 proteins with VIP > 1.0 distinguished baseline epileptic dogs (C0) from healthy adult controls (Fig. 4B).

**Fig. 4:**
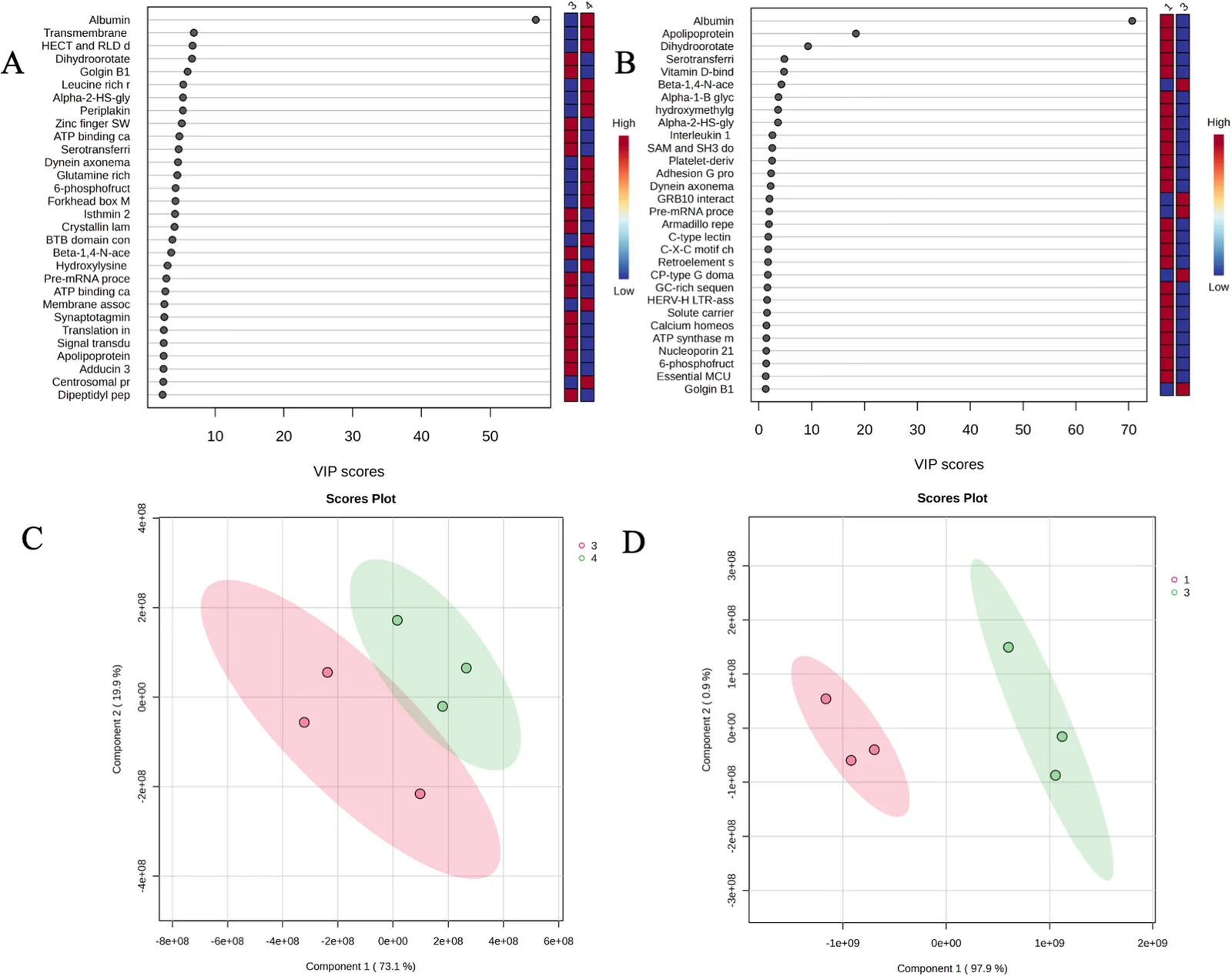
Variable importance in projection (VIP) scores and partial least squares discriminant analysis (PLS-DA) plots. (A) VIP scores of the 80 most significant proteins (VIP > 1.0) comparing the C60 vs. C0 groups. The color bar indicates the fold change direction (red: high/upregulated; blue: low/downregulated). (B) VIP scores of the 57 most significant proteins (VIP > 1.0) comparing adult controls vs. C0 groups. (C) PLS-DA score plot showing distinct separation and grouping of samples between the C60 (group 4, Green) and C0 (group 3, red) groups. Component 1 accounts for 73.1% of the variation. (D) PLS-DA scores plot showing separation between adult controls (group 3, red) and D0 (group 1, red/green markers are likely mislabeled in the source image but show clear group separation).

PLS-DA confirmed distinct proteomic signatures between C0 and C60 samples, with Component 1 accounting for 73.1% of the total variance (Fig. 4C). This clear separation validates that curcuminoid supplementation induced systematic and substantial alterations in the serum proteome.

#### 3.5.3 Downregulated proteins and pathway suppression

Protein–protein interaction (PPI) network analysis of downregulated proteins revealed significant suppression of key components within the mTOR signaling pathway, specifically Ras-related GTP-binding protein C (RRAGC) and regulatory-associated protein of mTOR (RPTOR) (Fig. 5A). In addition, Toll-like receptor 2 (TLR2), an innate immune sensor, was significantly downregulated.

**Fig. 5:**
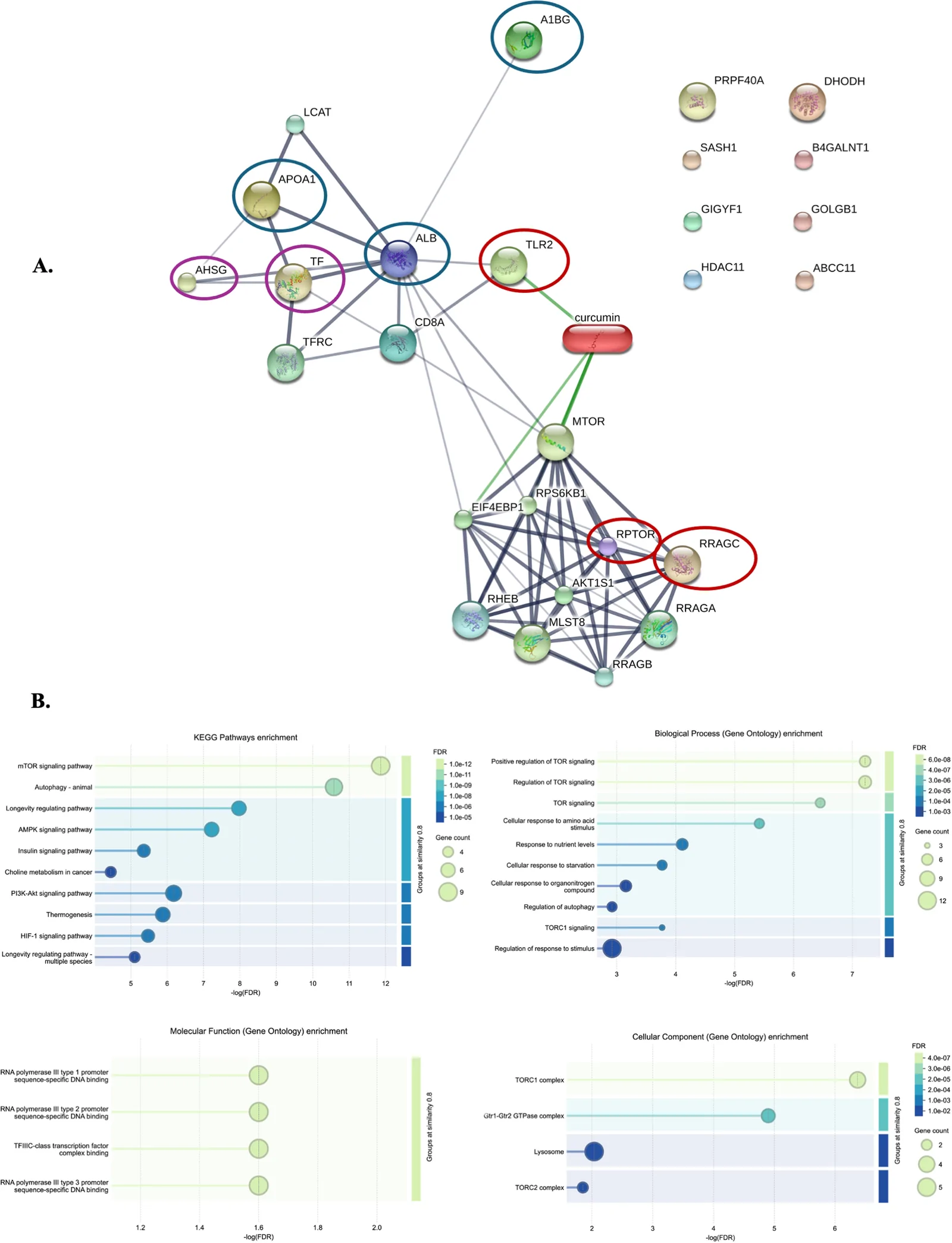
Protein–protein interaction (PPI) network and pathway enrichment analysis for downregulated proteins. (A) PPI network analysis showing the interaction of proteins that were significantly downregulated following curcuminoid supplementation (C60 vs. C0). Key downregulated proteins (red circles: Toll-like receptor 2 [TLR2], regulatory-associated protein of mTOR [RPTOR], and Ras-related GTP-binding protein C [RRAGC]; blue circles: albumin (ALB), apolipoprotein A1 (APOA1), and A1BG; purple circles: TF and alpha-2-HS-glycoprotein [AHSG]) are shown interacting with other cellular partners. The green line highlights the known anti-inflammatory/anticancer interaction between curcumin and TLR2. (B) Gene Ontology (GO) and Kyoto Encyclopedia of Genes and Genomes (KEGG) pathway enrichment analyses for downregulated proteins. Highly enriched pathways include the mammalian target of rapamycin (mTOR), autophagy, AMPK, and TLR signaling pathways, indicating a strong regulatory effect of curcumin on cellular growth, survival, and immune response.

Pathway enrichment analysis confirmed these findings, demonstrating significant suppression of the mTOR, autophagy, AMPK, and TLR signaling pathways (Fig. 5B). These results indicate that curcuminoid supplementation modulates cellular growth signaling and dampens innate immune activation (Table 4).

**Table 4:** Differentially expressed proteins between post-supplementation (day 60) and baseline (day 0) in dogs with primary epilepsy syndrome.

Protein ID	Protein name	Gene name	VIP score Ad -C0	VIP score C0-C60	-log10 *P*-value	FDR	Biological process	Cellular component	Molecular function
Seven common significant proteins in all groups									
A0A8C0P0P8	Albumin	ALB	70.672	56.626	3.7729	0.0004422	N/A	Extracellular space	Lipid binding and metal ion binding
A0A8I3MSG4	Apolipoprotein A1	APOA1	18.379	2.478	3.4289	0.0008051	Acylglycerol homeostasis, cholesterol homeostasis, glucocorticoid metabolic process, lipoprotein biosynthetic process, production involve in immune response, negative regulation of inflammatory response, positive regulation of phagocytosis, triglyceride homeostasis, and vitamin transport	Chylomicron, endocytic vesicle, extracellular vesicle, high-density lipoprotein particle, low-density lipoprotein particle, spherical high-density lipoprotein particle, and very-low-density lipoprotein particle	Apolipoprotein A-I receptor binding, chemorepellent activity, cholesterol binding, cholesterol transfer activity, high-density lipoprotein particle binding, high-density lipoprotein particle receptor binding, identical protein binding, and phospholipid binding
A0A8P0PDK7	GRB10 interacting GYF protein 1	GIGYF1	2.0279	1.3907	3.4104	0.0008327	N/A	N/A	N/A
A0A8I3MIF9	Alpha-1-B glycoprotein	A1BG	3.7148	1.3116	3.1766	0.0012642	Immune response-regulating signaling pathway	Extracellular region and plasma membrane	N/A
A0A8P0SGB8	Golgin B1	GOLGB1	1.3148	5.9638	2.8999	0.0021076	N/A	Golgi apparatus and membrane	N/A
A0A8I3NX92	SAM and SH3 domain containing 1	SASH1	2.5521	2.0037	2.8345	0.002378	N/A	Cytoplasm	N/A
A0A8P0N6S8	Zinc finger SWIM-type containing 9	ZSWIM9	1.0229	5.1166	2.1988	0.008322	N/A	N/A	Zinc ion binding
Downregulated proteins									
A0A8P0N657	Ras-related GTP binding C	RRAGC	0.2499	0.5494	3.0927	0.0014735	Cellular response to amino acid starvation, positive regulation of TORC1 signaling, and response to amino acid	Cytosol, lysosomal membrane, and nucleoplasm	GDP binding, GTP binding, GTPase activity, GTPase binding, molecular adaptor activity, and protein heterodimerization activity
A0A8I3NME4	Regulatory associated protein of MTOR	RPTOR	0.1961	0.4603	2.2811	0.0070355	Cellular response to amino acid stimulus, cellular response to glucose stimulus, cellular response to L-leucine, negative regulation of autophagy, positive regulation of cell growth, positive regulation of TOR signaling, regulation of autophagy, regulation of cell size and TORC1 signaling	Cytoplasm, cytoplasmic stress granule, cytosol, lysosomal membrane, nucleoplasm, serine/threonine protein kinase complex, and TORC1 complex	14-3-3 protein binding, enzyme-substrate adaptor activity, protein kinase activator activity, protein kinase binding, protein serine/threonine kinase inhibitor activity, protein-containing complex binding, protein-macromolecule adaptor activity, and small GTPase binding
A0A8C0S758	Histone deacetylase 11	HDAC11	0.2733	0.0151	4.9275	6.9269E-05	Chromatin organization and positive regulation of cell population proliferation	Chromatin, cytoplasm, nucleus, and protein-containing complex	DNA-binding transcription factor binding, histone deacetylase activity, and hydrolase activity
Q689D1	Toll-like receptor 2 precursor	TLR2	0.0227	0.0429	3.1321	0.0013777	Cellular response to diacyl bacterial lipopeptide, cellular response to triacyl bacterial lipopeptide, inflammatory response, innate immune response, and Toll-like receptor signaling pathway	Golgi apparatus, membrane raft, phagocytic vesicle membrane, plasma membrane, and receptor complex	NAD+ nucleosidase activity, cyclic ADP-ribose generating, signaling receptor activity, transmembrane signaling receptor activity, and triacyl lipopeptide binding
A0A8C0MFT0	T-cell surface glycoprotein CD8 alpha chain	CD8A	0.0762	0.0389	2.9129	0.0020598	Adaptive immune response, cell surface receptor signaling pathway, and T cell activation	External side of plasma membrane and receptor complex	N/A
Upregulated proteins									
A0A8C0M5Z2	Phosphoserine aminotransferase 1	PSAT1	0.0127	0.0534	3.0625	0.0015559	L-serine biosynthetic process	N/A	O-phospho-L-serine:2-oxoglutarate aminotransferase activity
A0A8P0NUZ2	Methylenetetrahydrofolate dehydrogenase	MTHFD1L	-	0.0372	4.5653	0.000121	Carboxylic acid biosynthetic process, formate metabolic process, and tetrahydrofolate interconversion	Mitochondrial matrix	ATP binding, formate-tetrahydrofolate ligase activity, and methylenetetrahydrofolate dehydrogenase (NADP+) activity
A0A8P0TT45	SH3 and multiple ankyrin repeat domains1	SHMT1	0.1215	1.9288	2.7382	0.0028506	Cell differentiation and nervous system development	Cytoplasm, plasma membrane, and postsynaptic density	N/A

#### 3.5.4 Upregulated proteins and metabolic reprogramming

PPI network analysis of upregulated proteins revealed a highly interconnected cluster of metabolic enzymes involved in one-carbon metabolism and amino acid biosynthesis (Fig. 6A). The key upregulated proteins included phosphoserine aminotransferase 1 (PSAT1) and methylenetetrahydrofolate dehydrogenase (MTHFD1L).

**Fig. 6:**
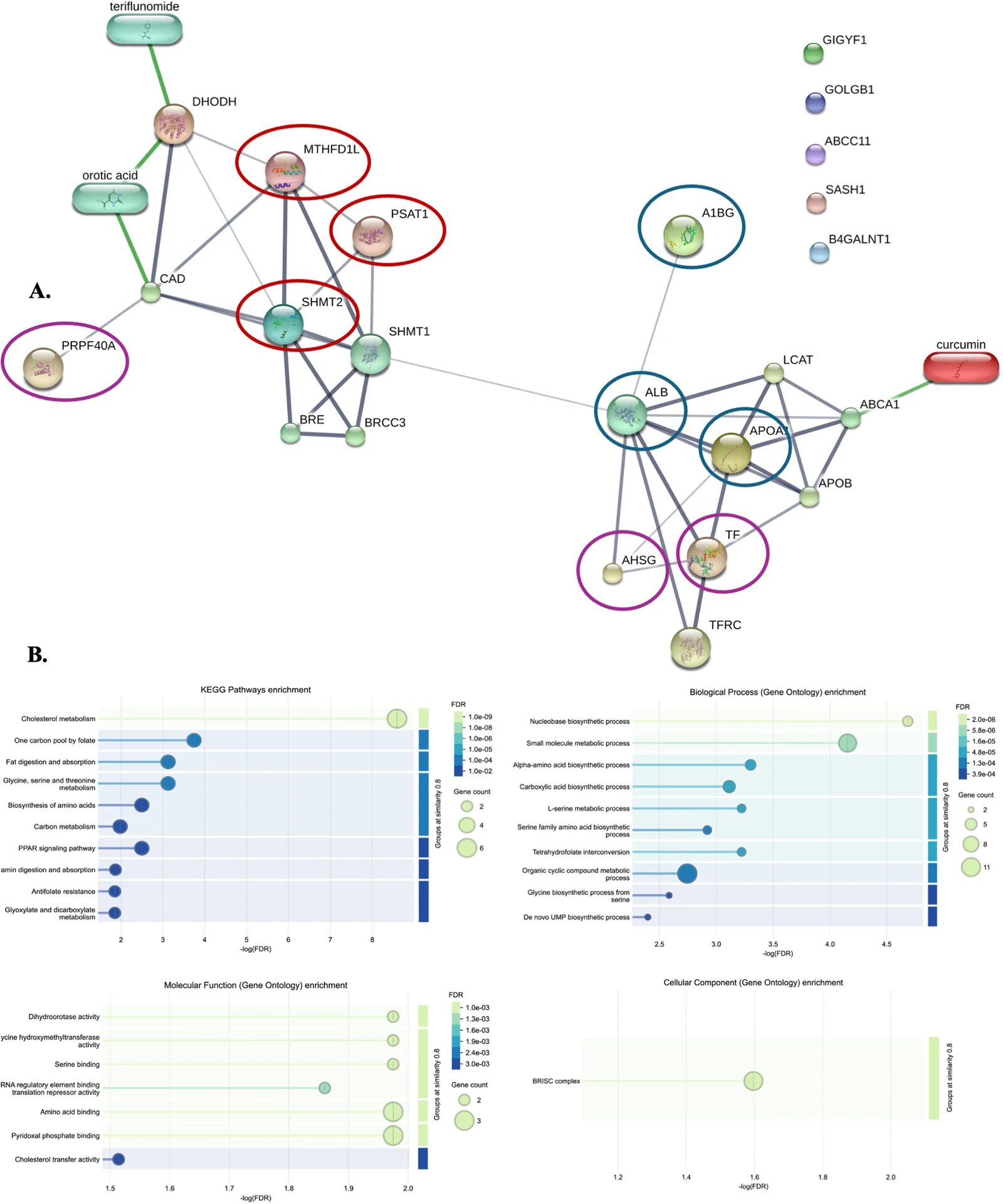
Protein–protein interaction (PPI) network and pathway enrichment analysis for upregulated proteins. (A) PPI network analysis showing the interaction of proteins that were significantly upregulated following curcuminoid supplementation (C60 vs. C0). Key upregulated proteins (red circles: phosphoserine aminotransferase 1 [PSAT1] and methylenetetrahydrofolate dehydrogenase [MTHFD1L]) are shown interacting within the folate and serine/glycine metabolic networks. (B). Gene Ontology (GO) and Kyoto Encyclopedia of Genes and Genomes (KEGG) pathway enrichment analyses for upregulated proteins. Highly enriched pathways include one-carbon pool by folate, glycine, serine, and threonine metabolism and de novo UMP biosynthetic process, suggesting metabolic reprogramming aimed at supporting cellular anabolism and methylation processes.

Pathway enrichment analysis identified significant upregulation of the one-carbon pool by folate, glycine/serine/threonine metabolism, and de novo UMP biosynthetic process (Fig. 6B). These findings suggest that curcuminoid supplementation promotes metabolic pathways supporting methylation, nucleotide synthesis, and cellular repair (Table 4).

Proteins are categorized into three groups: (1) seven common significant proteins identified across all analytical comparisons (VIP > 1.0 in both adults vs. C0 and C60 vs. C0); (2) downregulated proteins following curcuminoid supplementation, including mammalian target of rapamycin (mTOR) pathway components (Ras-related GTP-binding protein C [RRAGC] and regulatory-associated protein of mTOR [RPTOR]) and neuroinflammatory mediators (TLR2 and CD8A); and (3) upregulated proteins involved in one-carbon metabolism (methylenetetrahydrofolate dehydrogenase [MTHFD1L]) and serine biosynthesis (phosphoserine aminotransferase 1 [PSAT1] and SHMT1). VIP scores, −log_10_ *P*-values, false discovery rates (FDRs), and functional annotations are provided. Proteomic analysis was performed using nano-liquid chromatography–tandem mass spectrometry with MaxQuant and Perseus software platforms.*3.5.5. Convergent Biomarkers*

The analysis of significantly altered proteins in disease state and treatment response identified six proteins with VIP ≥ 1.0 in both comparisons (adults vs. C0 and C60 vs. C0) (Table 5), including dihydroorotate dehydrogenase (DHODH), serotransferrin, and alpha-2-HS-glycoprotein. DHODH, which is involved in pyrimidine biosynthesis, exhibited high-VIP scores in disease (VIP = 9.3) and treatment (VIP = 6.6) comparisons, suggesting its potential as a convergent biomarker linking epilepsy pathology to therapeutic response.

**Table 5:** Shared significant proteins identified by VIP analysis in adult control vs. baseline (adults vs. C0) and post-supplementation vs. baseline (C60 vs. C0) comparisons.

Protein ID	Protein name	Gene name	VIP score Adult vs C0	VIP score C0 vs C60	Biological process	Cellular component	Molecular function
A0A8I3N262	Dihydroorotate dehydrogenase (quinone), mitochondrial	DHODH	9.3034	6.6252	“De novo” pyrimidine nucleobase biosynthetic process, “de novo” UMP biosynthetic process, proteolysis, and pyrimidine ribonucleotide biosynthetic process	Extracellular region and mitochondrial inner membrane	Dihydroorotate dehydrogenase activity, hemoglobin binding, and serine-type endopeptidase activity
A0A8C0MKY4	Serotransferrin	LOC477072	4.8327	4.6512	Intracellular iron ion homeostasis and negative regulation of multicellular organismal process	Extracellular space	Ferric iron binding
A0A8I3Q5N4	Beta-1,4-N-acetyl-galactosaminyltransferase	B4GALNT4	4.2755	3.578	N/A	Golgi cisterna membrane	Acetylgalactosaminyltransferase activity
A0A8I3PIK8	Alpha-2-HS-glycoprotein	AHSG	3.623	5.2995	Acute-phase response, positive regulation of phagocytosis, and regulation of inflammatory response	Extracellular matrix, extracellular region, extracellular space, and Golgi apparatus	Cysteine-type endopeptidase inhibitor activity and endopeptidase inhibitor activity
A0A8I3NHE8	Pre-mRNA processing factor 40 homolog A	PRPF40A	3.623	2.8731	mRNA cis splicing via spliceosome	Nuclear matrix and nuclear speck	N/A
A0A8C0MYW8	ATP binding cassette subfamily C member 11	ABCC11	1.2196	2.7253	N/A	Membrane	ABC-type transporter activity, ATP binding, and ATP hydrolysis activity

Six proteins with VIP scores > 1.0 in both comparison groups are presented with UniProt identifiers, gene names, VIP scores, and functional annotations (biological process, cellular component, and molecular function). These proteins represent conserved metabolic and structural alterations associated with epilepsy pathophysiology and curcuminoid intervention.

## 4 Discussion

This study provides the first integrated clinical and proteomic assessment of curcuminoid supplementation in dogs with PES. In this uncontrolled pilot study (n = 8), curcuminoid supplementation was safe, well tolerated, and yielded a clinical response rate of 50% in dogs with PES; however, the absence of a concurrent control group limits interpretation to preliminary evidence of an association rather than definitive evidence of efficacy. Importantly, proteomic analysis identified signatures consistent with a dual mechanism: (1) potential neuroprotection through enhanced antioxidant capacity and suppression of pro-epileptogenic mTOR and TLR2 signaling pathways and (2) metabolic reprogramming via upregulation of one-carbon metabolism and serine biosynthesis pathways in the peripheral compartment, which may serve as systemic surrogate indicators of neuroprotective processes (Luo et al., 2026; Mattsson-Carlgren, 2025).

### 4.1 Clinical efficacy and safety profile

The 50% clinical response rate observed in this preliminary is comparable to those reported for second-line AEDs in canine epilepsy (Charalambous et al., 2014). Notably, curcuminoid supplementation demonstrated an excellent safety profile with no serious adverse events, contrasting favorably with the hepatotoxicity, sedation, and hematological abnormalities frequently associated with conventional AEDs (Charalambous et al., 2024).

The finding that clinical improvement occurred in only 50% of dogs suggests potential heterogeneity in therapeutic response, which may be related to individual differences in curcuminoid bioavailability, genetic polymorphisms affecting drug metabolism, or variations in underlying epilepsy pathophysiology. Future studies with larger sample sizes are needed to identify the predictive biomarkers of treatment response.

### 4.2 Restoration of redox homeostasis

The significant improvement in the GSH/GSSG ratio (*P* = 0.046) provides evidence consistent with curcuminoid-associated enhancement of antioxidant capacity. GSH is a critical antioxidant in both the peripheral and central compartments; however, serum GSH/GSSG measured here reflects the systemic redox status and cannot be equated directly with intracellular brain GSH, which is regulated independently across the blood-brain barrier. Nonetheless, systemic oxidative stress is itself a recognized contributor to epilepsy pathophysiology, and its amelioration may indirectly reflect or support neuronal redox balance (Parsons et al., 2022; Zimmerman et al., 2026). Curcuminoids enhance GSH synthesis by activating the Nrf2 pathway, which upregulates the expression of glutamate–cysteine ligase (the rate-limiting enzyme in GSH biosynthesis) (Shahcheraghi et al., 2022).

Importantly, improvement in the GSH/GSSG ratio was observed in clinical responders and nonresponders, suggesting that antioxidant effects alone may be insufficient for seizure control in all patients. This finding underscores the multifactorial nature of epilepsy pathophysiology and highlights the importance of concurrent modulation of inflammatory and metabolic pathways for optimal therapeutic efficacy.

The selective improvement in GSH/GSSG ratio without concurrent MDA change is mechanistically consistent with curcuminoids functioning primarily as Nrf2 activators, a pathway that rapidly enhances glutathione based antioxidant defenses without necessarily reversing preexisting membrane lipid peroxidation. Curcuminoids activate the Nrf2/Keap1/ARE signaling axis at tissue concentrations sufficient to upregulate glutamate cysteine ligase, glutathione reductase, and glutathione peroxidase, thereby improving the GSH/GSSG ratio, while remaining below the threshold required to directly suppress lipid peroxidation within membrane compartments (Mishra et al., 2019; Serafini et al., 2019; Shahcheraghi et al., 2022). Unlike MDA, which represents slowly reversing cumulative structural lipid damage requiring both cessation of peroxidation chain reactions and metabolic clearance of preexisting lipid adducts (Tsikas, 2017), the GSH/GSSG ratio constitutes a dynamically regulated thiol redox index that responds more proximally and sensitively to curcuminoid antioxidant activity, a pattern consistent with clinical evidence demonstrating that hemodialysis patients receiving curcumin 1 g/day for 12 weeks showed significantly preserved glutathione peroxidase activity and increased catalase activity, yet MDA and inflammatory markers were not significantly reduced versus placebo (Rodrigues et al., 2021).

### 4.3 Suppression of mTOR signaling: a central neuroprotective mechanism

The downregulation of key mTOR pathway components, including RPTOR (also known as Raptor) and RRAGC, was one of the most notable proteomic findings. mTOR is a serine/threonine kinase that integrates nutrient, energy, and growth factor signals to regulate cell growth, proliferation, and metabolism. mTOR forms two distinct complexes: mTORC1 (containing RPTOR) and mTORC2. RPTOR is essential for mTORC1 assembly and substrate recruitment, whereas RRAGC regulates amino-acid-dependent mTORC1 activation. mTORC1 signaling hyperactivation is a well-established driver of epileptogenesis. Excessive mTORC1 activity promotes abnormal dendritic spine morphology, dysregulated protein synthesis, impaired autophagy, and enhanced neuronal excitability (Boff et al., 2025; Cullen et al., 2024; Yang et al., 2025). mTOR inhibitors (e.g., rapamycin and everolimus) demonstrated potent anticonvulsant effects in experimental models and human epilepsy syndromes, particularly tuberous-sclerosis-complex-associated epilepsy (Drion et al., 2016; Zhao et al., 2023).

The finding that curcuminoid supplementation downregulates RPTOR and RRAGC suggests that curcuminoids exert anticonvulsant effects, at least in part, through mTORC1 inhibition. This mechanism is consistent with previous studies demonstrating that curcumin suppresses mTOR signaling in cancer and neurodegenerative disease models (Drion et al., 2016; Drion et al., 2019; Khayatan et al., 2025). Serum downregulation of RPTOR and RRAGC indicates systemic mTORC1 suppression, which cannot be extrapolated to neuronal tissue across the blood-brain barrier. These findings are therefore interpreted as peripheral surrogate indicators of a pathway critically implicated in epileptogenesis.

### 4.4 Suppression of TLR2-mediated inflammation

The downregulation of TLR2 observed in this study provides proteomic evidence consistent with curcuminoid-associated modulation of inflammatory pathways. TLR2 is a pattern recognition receptor expressed on microglia, astrocytes, and neurons that detects damage-associated molecular patterns released during seizure-induced neuronal injury. TLR2 activation triggers downstream NF-κB and MAPK signaling cascades, resulting in the production of proinflammatory cytokines (IL-1β, TNF-α, and IL-6) and chemokines that perpetuate neuroinflammation and lower the seizure threshold (Medel-Matus et al., 2017; Sanz & Garcia-Gimeno, 2020). Elevated TLR2 expression has been documented in brain tissue from patients with temporal lobe epilepsy and experimental epilepsy models, and TLR2 knockout mice have been found to exhibit reduced seizure susceptibility (Medel-Matus et al., 2017).

The finding that curcuminoid supplementation downregulates serum TLR2 provides proteomic evidence of TLR2 pathway modulation that may indirectly reflect central nervous system neuroinflammatory signaling. Peripheral TLR2 may serve as a clinically accessible surrogate for systemic neuroinflammatory burden, as circulating soluble TLR2 has been shown to correlate with pro-inflammatory molecules (IL-6, TNF-α, IL-1R) during systemic inflammatory activation and with neurological outcome in ischemic stroke (Brea et al., 2011; Mothapo et al., 2017; Ten Oever et al., 2014). Curcuminoids are known to inhibit TLR2 signaling through multiple mechanisms, including direct binding to TLR2, interference with TLR2 dimerization, and suppression of downstream NF-κB activation (Shuto et al., 2010; Tu et al., 2014).

The concurrent suppression of the mTOR and TLR2 pathways is particularly noteworthy as these pathways exhibit extensive crosstalk. TLR2 activation can stimulate mTORC1 activity, whereas mTORC1 hyperactivation enhances inflammatory cytokine production (Bono et al., 2022; Imanishi et al., 2020). Thus, the curcuminoid-mediated inhibition of both pathways may produce synergistic neuroprotective effects.

The absence of changes in serum IL-6 and IL-10 despite TLR2 downregulation is not contradictory. TLR2 suppression reflects modulation of an upstream pattern recognition receptor that reduces the capacity for inducible pro-inflammatory activation rather than resting cytokine output, as receptor-level tolerance can be established independently of paracrine cytokine transfer, and negative regulators of TLR2 signaling reduce NF-κB-driven inducible responses without altering basal cytokine concentrations (Lehner et al., 2001; Murphy et al., 2015; Wang et al., 2002). In clinically stable inter-ictal dogs, serum IL-6 and IL-10 are expected to remain at homeostatic setpoints regardless of upstream TLR2 modulation. Furthermore, serum cytokine measurements reflect the systemic compartment and may not capture neuroinflammatory dynamics within the CNS microenvironment.

### 4.5 Upregulation of one-carbon metabolism: supporting neuronal repair

A novel and unexpected finding of this study was the upregulation of MTHFD1L, a key enzyme in mitochondrial one-carbon metabolism. MTHFD1L catalyzes the conversion of 10-formyltetrahydrofolate to tetrahydrofolate and formate, providing one-carbon units essential for purine and thymidine synthesis, mitochondrial protein translation, and epigenetic regulation through DNA and histone methylation.

One-carbon metabolism is critical for maintaining cellular biosynthetic capacity, particularly during periods of high metabolic demand, including post seizure neuronal repair. Formate produced by MTHFD1L is exported from the mitochondria to the cytosol, where it supports nucleotide synthesis for DNA repair and cell proliferation. In addition, one-carbon metabolism generates NADPH, which is essential for GSH regeneration and maintenance of redox homeostasis (Guan et al., 2024; Zhao et al., 2021).

According to emerging evidence, impaired one-carbon metabolism may contribute to epilepsy pathophysiology. Genetic variants in folate metabolism genes, including methylenetetrahydrofolate reductase (*MTHFR*), are associated with increased epilepsy risk (Colliva et al., 2019; Scher et al., 2011; Zou et al., 2022). Conversely, folate supplementation has been shown to reduce seizure frequency in some patients (De Luca et al., 2022; Morrell, 2002).

MTHFD1L upregulation in serum suggests enhanced systemic biosynthetic capacity that may indirectly support post-seizure recovery, though direct inference of neuronal repair cannot be drawn from serum proteomics alone. This metabolic reprogramming may complement the neuroprotective effects of mTOR and TLR2 suppression, creating a favorable cellular environment for recovery from seizure-induced damage.

### 4.6 Upregulation of serine biosynthesis: restoring neurotransmitter balance

The upregulation of PSAT1, a key enzyme in the serine biosynthesis pathway, is closely related to one-carbon metabolism. PSAT1 catalyzes the conversion of 3-phosphohydroxypyruvate to phosphoserine, the penultimate step in de novo serine synthesis. Serine is a nonessential amino acid with critical roles in neurotransmitter synthesis, lipid metabolism, and one-carbon metabolism.

In the central nervous system, serine serves as the precursor for D-serine and glycine, which are co-agonists of the N-methyl-D-aspartate (NMDA) receptor. D-serine, synthesized by serine racemase in astrocytes, is essential for NMDA receptor activation and synaptic plasticity. Glycine is the primary inhibitory neurotransmitter in the spinal cord and brainstem and modulates NMDA receptor function. The dysregulation of serine metabolism has been implicated in neurological disorders, including epilepsy. Genetic defects in serine biosynthesis enzymes cause severe neurological phenotypes characterized by intractable seizures, microcephaly, and developmental delay (Klatte et al., 2013; Ma et al., 2019; Shen et al., 2022).

PSAT1 upregulation in serum following curcuminoid supplementation suggests enhanced systemic serine biosynthesis capacity, reflecting peripheral metabolic reprogramming following curcuminoid supplementation. As serine is a key substrate in one-carbon metabolism and amino acid homeostasis, its increased systemic availability may contribute to the broader metabolic adaptations observed in this study.

### 4.7 Convergence with teriflunomide: metabolic modulation as an antiepileptic strategy

The observed metabolic phenotype—characterized by mTORC1 suppression, enhanced one-carbon metabolism, and upregulated serine biosynthesis—shares functional similarities with teriflunomide (an immunomodulatory drug approved for multiple sclerosis that has shown promise in epilepsy treatment). Teriflunomide inhibits DHODH, the rate-limiting enzyme in de novo pyrimidine synthesis, thereby restricting the proliferation of activated immune cells and reducing neuroinflammation (Palmer, 2010; Wostradowski et al., 2016).

Importantly, DHODH inhibition also modulates neuronal metabolism. Teriflunomide reduces the metabolic capacity of hyperexcitable neurons by limiting pyrimidine availability, effectively lowering the seizure threshold (Gallagher, 2019; Uytterhoeven et al., 2019). Moreover, it activates Nrf2 signaling, enhancing antioxidant defenses by upregulating heme oxygenase-1 (HO-1) and other cytoprotective enzymes (Bar-Or et al., 2014; Brandes & Gray, 2020).

Although curcuminoids do not directly inhibit DHODH, the upregulation of PSAT1 and MTHFD1L suggests compensatory enhancement of alternative biosynthetic pathways. Curcuminoids may provide nucleotide precursors while simultaneously restricting the metabolic fuel available for neuroinflammation by increasing serine and one-carbon unit availability. The suppression of mTORC1 signaling parallels the metabolic set-point reduction achieved by DHODH inhibition, preventing hypersynchronous neuronal activity (Cullen et al., 2024; Uytterhoeven et al., 2019).

This metabolic modulation, combined with GSH/GSSG restoration—analogous to teriflunomide’s Nrf2/HO-1 pathway activation—positions curcuminoids as broad-spectrum metabolic-immune regulators that disrupt the self-perpetuating cycle of oxidative stress, neuroinflammation, and hyperexcitability underlying epileptogenesis.

### 4.8 Study limitations

This study has several important limitations that must be acknowledged. First, the small sample size (n = 8) limits the statistical power and generalizability of the findings. The open-label design without a placebo control group precludes the definitive exclusion of placebo effects or spontaneous remission, both of which are recognized phenomena in canine epilepsy. The observed clinical response rate and significant biochemical changes provide preliminary evidence of an association between supplementation and clinical improvement. However, large-scale, double-blind, randomized controlled trials are essential to validate these findings. The correlation between individual proteomic changes and seizure frequency or severity was not investigated, limiting direct interpretation of clinical significance. Future studies with larger sample sizes should incorporate correlation analyses between changes in key protein abundances (RPTOR, RRAGC, TLR2, MTHFD1L, and PSAT1) and seizure outcomes to determine whether these signatures serve as predictive biomarkers of therapeutic response.

Second, whether the observed proteomic changes are specific to curcuminoid intervention or reflect natural variability in epilepsy pathophysiology could not be determined because of the lack of a control group receiving placebo supplementation. Future studies should include parallel placebo-treated control groups with identical sampling and analytical protocols.

Third, serum proteomic profiling, while informative, may not fully reflect changes occurring in the central nervous system. Cerebrospinal fluid proteomics or brain tissue analysis would provide a more direct assessment of neurological effects, although such approaches are not feasible in client-owned companion animals. Nevertheless, serum biomarkers may serve as accessible surrogate markers for central nervous system processes, particularly given the systemic nature of inflammation and oxidative stress in epilepsy. The blood-brain barrier imposes a fundamental constraint on the interpretation of serum and plasma proteomic data: proteins identified in circulation do not necessarily cross into the central nervous system compartment, and differential protein expression in the blood cannot be used to infer equivalent changes in brain parenchyma, hippocampus, or synaptic microenvironments. The observed serum/plasma proteomic changes are therefore best interpreted as systemic and peripheral metabolic and immune consequences of epilepsy and its treatment, and as potential indirect surrogate markers for central nervous system processes.

Fourth, plasma or serum curcuminoid concentrations were not measured to assess bioavailability and pharmacokinetics. Curcumin is known to have poor oral bioavailability because of rapid metabolism and elimination. However, bioavailability-enhanced formulations are available. Future studies should include pharmacokinetic assessments to correlate curcuminoid exposure with clinical and molecular outcomes and optimize dosing regimens.

Fifth, the 60-day intervention period may be insufficient to capture the long-term effects of curcuminoid supplementation on epilepsy progression and disease modification. Extended follow-up studies are needed to determine whether curcuminoids exert disease-modifying effects beyond symptomatic seizure control.

Sixth, dietary composition was not formally standardized or recorded, which represents a potential confounding variable for the interpretation of one-carbon metabolic pathway findings.

Finally, the validation of key proteomic findings through orthogonal methods (e.g., Western blot analysis, ELISA, or immunohistochemistry) would strengthen confidence in the identified biomarkers. Although pathway-level convergence provides supporting evidence, direct confirmation of individual protein expression changes is desirable.

## 5 Conclusions

This open-label pilot study provides preliminary evidence of the safety and potential antiseizure activity of curcuminoid supplementation as adjunctive therapy for canine PES. Given the uncontrolled design and small sample size (n = 8), the 50% clinical response rate and improvement in redox homeostasis (GSH/GSSG ratio, *P* = 0.046) are best interpreted as evidence of an association rather than definitive evidence of efficacy. Proteomic analysis identified signatures consistent with suppression of mTORC1 and TLR2 pathways alongside upregulation of one-carbon metabolism and serine biosynthesis, suggesting potential immune modulation and neuronal repair support. As peripheral findings, these are indicative of but do not directly demonstrate neuroprotection, which requires confirmation via CSF analysis, EEG, or histopathology. Collectively, these results support the rationale for curcuminoids as metabolic-immune modulators and justify larger, prospective, randomized controlled trials.

The identified biomarkers (RPTOR, RRAGC, PSAT1, MTHFD1L, and TLR2) provide a mechanistic framework for therapeutic monitoring and may be used as convergent metabolic targets for epilepsy treatment. However, validation through large-scale, double-blind, randomized controlled trials is essential to confirm efficacy, optimize dosing, and identify responder profiles.

These findings support the integration of curcuminoids into multimodal epilepsy management strategies in veterinary medicine, with potential translational implications for human epilepsy therapy.

## Ethical statement

This study was conducted at Prasu Arthorn Animal Hospital, Faculty of Veterinary Science, Mahidol University, and approved by the Mahidol University Institute Animal Care and Use Committee (Protocol No. MUVS-2020-08-37).

## Ethics declaration

This study was conducted in accordance with the ARRIVE (Animal Research: Reporting of In Vivo Experiments) guidelines. This study was approved by the Mahidol University Institute Animal Care and Use Committee of the Faculty of Veterinary Science. (Approval No. Protocol No. MUVS-2020-08-37)

## Funding sources

This work was supported by Faculty of Veterinary Science, Mahidol University.

## CRediT authorship contribution statement

**Boonrat Chantong:** Writing – review & editing, Writing – original draft, Visualization, Validation, Supervision, Resources, Project administration, Methodology, Investigation, Funding acquisition, Formal analysis, Data curation, Conceptualization. **Nutnicha Prakaikowit:** Writing – review & editing, Investigation, Data curation. **Wanita Sangapol:** Writing – review & editing, Investigation, Data curation. **Sirakarn Chatampaiwong:** Writing – review & editing, Investigation, Data curation. **Sekkarin Ploypetch:** Writing – review & editing, Writing – original draft, Visualization, Resources, Methodology, Investigation, Formal analysis, Data curation, Conceptualization. **Sataporn Phochantachinda:** Writing – review & editing, Methodology, Investigation, Formal analysis, Data curation, Conceptualization. **Duangthip Chatchaisak:** Writing – review & editing, Writing – original draft, Visualization, Validation, Supervision, Resources, Project administration, Methodology, Investigation, Funding acquisition, Formal analysis, Data curation, Conceptualization.

## Declaration of competing interest

The authors declare the following financial interests/personal relationships which may be considered as potential competing interests:

Duangthip Chatchaisak reports equipment, drugs, or supplies was provided by Kaewmangkorn Pharmacy Co., Ltd., Thailand. If there are other authors, they declare that they have no known competing financial interests or personal relationships that could have appeared to influence the work reported in this paper.

## Data Availability

The MS proteomics data have been deposited to the ProteomeXchange Consortium via the PRIDE partner repository with the dataset identifier PXD076578 to be completed.
